# Preserved acute pain and impaired neuropathic pain in mice lacking protein interacting with C Kinase 1

**DOI:** 10.1186/1744-8069-7-11

**Published:** 2011-02-03

**Authors:** Wei Wang, Ronald S Petralia, Kogo Takamiya, Jun Xia, Yun-Qing Li, Richard L Huganir, Yuan-Xiang Tao, Myron Yaster

**Affiliations:** 1 Department of Anesthesiology and Critical Care Medicine, Johns Hopkins University School of Medicine, Baltimore, Maryland 21205, USA; 2 Department of Anatomy, Histology and Embryology, K. K. Leung Brain Research Centre, the Fourth Military Medical University, Xi'an, 710032, PR China; 3 Laboratory of Neurochemistry, National Institute of Deafness and Other Communication Disorders, National Institutes of Health, Bethesda, Maryland 20892, USA; 4 Department of Neuroscience, Johns Hopkins University School of Medicine, Baltimore, Maryland 21287, USA; 5 Department of Biochemistry, The Hong Kong University of Science and Technology, Clear Water Bay, Kowloon, Hong Kong, PR China

## Abstract

Protein interacting with C Kinase 1 (PICK1), a PDZ domain-containing scaffolding protein, interacts with multiple different proteins in the mammalian nervous system and is believed to play important roles in diverse physiological and pathological conditions. In this study, we report that PICK1 is expressed in neurons of the dorsal root ganglion (DRG) and spinal cord dorsal horn, two major pain-related regions. PICK1 was present in approximately 29.7% of DRG neurons, most of which were small-less than 750 μm^2 ^in cross-sectional area. Some of these PICK1-positive cells co-labeled with isolectin B4 or calcitonin-gene-related peptide. In the dorsal horn, PICK1 immunoreactivity was concentrated in the superficial dorsal horn, where it was prominent in the postsynaptic density, axons, and dendrites. Targeted disruption of PICK1 gene did not affect basal paw withdrawal responses to acute noxious thermal and mechanical stimuli or locomotor reflex activity, but it completely blocked the induction of peripheral nerve injury-induced mechanical and thermal pain hypersensitivities. PICK1 appears to be required for peripheral nerve injury-induced neuropathic pain development and to be a potential biochemical target for treating this disorder.

## Introduction

Neurotransmission requires spatial and functional assembly of signal transduction machinery at the plasma membrane. The postsynaptic density, an electron-dense cytoskeletal structure beneath the plasma membrane of excitatory synapses, is one site where receptors, channels, and effectors organize to mediate signaling [1]. The postsynaptic density contains membrane proteins such as AMPA receptor (AMPAR) subunits and NMDA receptor subunits, signal transduction molecules such as protein kinase C alpha (PKCα) and neuronal nitric oxide synthase, and scaffolding proteins [1,2]. Most scaffolding proteins contain one or more PDZ (PSD-95/Dlg/ZO-1) amino acid domains [1,3,4]. Through PDZ domain interaction, they assemble intracellular signaling complexes around synaptic receptors, regulate synaptic and non-synaptic receptor trafficking and functions, and participate in many physiological and pathological processes triggered via the activation of synaptic receptors [1,3-6].

Protein interacting with C Kinase 1 (PICK1), a PDZ domain-containing scaffolding protein that is enriched in the postsynaptic density, initially was reported to interact with PKCα [7] and subsequently was found to bind to synaptic AMPAR subunit GluR2 in central neurons [8-10]. We recently reported that, via its PDZ domain, PICK1 interacts with GluR2 and PKCα, recruits intracellular PKCα to synaptic GluR2, and leads to GluR2 phosphorylation at Ser880 [11-14]. This phosphorylation disrupts the interaction between synaptic GluR2 and the anchor protein AMPAR-binding protein/glutamate receptor-interacting protein; promotes synaptic GluR2 internalization; and increases synaptic GluR2-lacking, Ca^2+ ^permeable AMPARs in dorsal horn neurons [11-14]. In addition, we have shown previously that preventing dorsal horn GluR2 internalization through targeted disruption of PICK1 gene attenuates complete Freund's adjuvant (CFA)-induced pain hypersensitivity during the maintenance period [14]. These findings suggest that spinal PICK1 may participate in the maintenance of persistent inflammatory pain by promoting dorsal horn GluR2 internalization. However, the expression and distribution of PICK1 in the pain-related regions of the nervous system have not been carefully studied. In addition, CFA-induced inflammatory pain and nerve injury-induced neuropathic pain might share some intracellular signaling pathways in their central mechanisms [15], but whether PICK1 is also involved in the development and maintenance of nerve injury-induced persistent neuropathic pain is unknown.

In the present study, we first characterized the expression and distribution of PICK1 in two major pain-related regions, the dorsal root ganglion (DRG) and spinal cord dorsal horn. Then, we addressed the role of PICK1 in neuropathic pain induced by fifth lumbar (L_5_) spinal nerve ligation (SNL) and distal transection. Finally, we examined whether peripheral nerve injury, like peripheral inflammation, induces dorsal horn GluR2 internalization and whether this induction requires spinal PICK1 under neuropathic pain conditions.

## Materials and methods

### Animal preparation

Male mice (10-12 weeks old) and male Sprague-Dawley rats (225-250 g) were housed on a standard 12-h light/dark cycle, with water and food pellets available *ad libitum*. PICK1 knockout (KO) mice (C57BL/6J genetic background) were generated as described previously [16]. Male PICK1 KO mice and wild-type (WT) littermates were obtained by interbreeding PICK1 heterozygous mice. To minimize intra- and inter-individual variability of behavioral outcome measures, animals were trained for 1-2 days before behavioral testing was performed. Animal experiments were conducted with the approval of the Animal Care and Use Committee at Johns Hopkins University and were consistent with the ethical guidelines of the National Institutes of Health and the International Association for the Study of Pain. All efforts were made to minimize animal suffering and to reduce the number of animals used. The experimenters were blind to mouse genotype during the behavioral testing.

### Intrathecal administration of PICK1 antisense (AS) oligodeoxynucleotide (ODN)

To further confirm the results from PICK1 KO mice, we used a PICK1 AS ODN approach to knock down PICK1 in spinal cord and DRG of rats. Rats were used because the subarachnoid space of mice is too small to implant the intrathecal (i.th.) polyethylene (PE-10) catheter that is required for repeated ODN injections. The design of PICK1 AS ODN, its control missense (MS) ODN, and their i.th. administration have been described in our previous study [14]. Briefly, an i.th. PE-10 catheter was inserted into the subarachnoid space at the rostral level of the spinal cord lumbar enlargement segments through an incision at the atlanto-occipital membrane [14,17,18]. One week later, rats that showed no neurological deficits received an i.th. injection of saline (10 μl; control), AS ODN (10 μg/10 μl), or MS ODN (10 μg/10 μl) once daily for 4 days. On the second day after ODN injection, an L_5 _SNL model of neuropathic pain or sham surgery was performed as described below.

### Neuropathic pain model

An L_5 _SNL model of neuropathic pain was produced in mice and rats according to the method described previously [6,18,19]. In brief, animals were anesthetized with isoflurane, and a dorsolateral skin incision was made on the lower back. The sixth lumbar transverse process was identified and freed of its muscle attachment and then removed. The underlying fifth lumbar nerve root was isolated, ligated with a silk suture (6-0 in mice; 3-0 in rats), and transected just distal to the ligature. After appropriate hemostasis, the skin and muscle layers were closed with a silk suture. In the sham group, the surgical procedure was identical to that described above, except that the spinal nerve was not ligated or transected. Paw withdrawal responses to thermal and mechanical stimuli on the ipsilateral and contralateral sides were examined 1 day before and then at various time point after SNL or sham surgery as described below.

### Behavioral testing

Tail-flick assay: The mouse (5 WT and 5 KO mice) was placed in a Plexiglas cylinder. A tail-flick apparatus (Model 33B Tail Flick Analgesia Meter, IITC Life Science, Woodland Hills, CA, USA) with a radiant heat source connected to an automatic timer was used to assess the analgesic response. A cut-off time latency of 10 sec was used to avoid tissue damage to the tail. Tail-flick latencies were measured as the time required to induce a tail flick after applying radiant heat to the skin of the tail. Each trial was repeated five times at 5-min intervals.

Paw withdrawal responses to noxious thermal stimuli: The mouse (10 WT and 10 KO mice) or rat (15 rats) was placed in a Plexiglas chamber on a glass plate above a light box. Radiant heat from Model 336 Analgesia Meter (IITC Life Science) was applied by aiming a beam of light through a hole in the light box through the glass plate to the middle of the plantar surface of each hind paw. When the animal lifted its foot, the light beam automatically shut off. The length of time between the start of the light beam and the foot lift was defined as the paw withdrawal latency. Each trial was repeated five times at 5-min intervals for each side. A cut-off time of 20 sec was used to avoid tissue damage to the hind paw.

Paw withdrawal responses to repeated mechanical stimuli: The mouse (10 WT and 10 KO mice) or rat (15 rats) was placed in a Plexiglas chamber on an elevated mesh screen. In mice, two calibrated von Frey monofilaments (0.24 and 4.33 mN; Stoelting Co., Wood Dale, IL, USA) were employed. Each von Frey filament was applied to the hind paw for approximately 1 sec, and each trial was repeated 10 times to both hind paws. In rats, a single trial of mechanical stimuli consisted of eight applications of a calibrated von Frey filament (8.01 mN) within a 2-3-sec period. Each trial was repeated 10 times at 3-min intervals on each hind paw. The occurrence of paw withdrawal in each of these 10 trials was expressed as a percent response frequency [(number of paw withdrawals/10 trials) × 100 = % response frequency], and this percentage was used as an indication of the amount of paw withdrawal.

### Locomotor function testing

The following tests were performed with the experimenter blind to the mouse genotype (5 WT and 5 KO mice): (1) Placing reflex: the experimenter held the animal's hind limbs slightly lower than the forelimbs and brought the dorsal surfaces of the hind paws into contact with the edge of a table. The experimenter recorded whether the hind paws were placed on the table surface reflexively. (2) Grasping reflex: the experimenter placed the animal on a wire grid and recorded whether the hind paws grasped the wire on contact. (3) Righting reflex: the experimenter placed the animal's back on a flat surface and noted whether it immediately assumed the normal upright position. Scores for placing, grasping, and righting reflexes were based on counts of each normal reflex exhibited in five trials. In addition, the animal's general behaviors, including spontaneous activity, were observed.

### Immunohistochemistry

The mice (6 WT and 1 KO mouse) were deeply anesthetized with isoflurane and perfused with 4% paraformaldehyde in phosphate buffer (0.1 M, pH 7.4). The L_4-5 _spinal cord segments and DRGs were harvested, post-fixed in the same fixative solution for 2-4 h, and cryoprotected by immersion in 30% sucrose overnight at 4°C. The transverse sections (20 μm) were cut on a cryostat, and four sets of sections from WT mice were collected. PICK1 immunohistochemical staining alone was performed on the first set of sections. Briefly, the sections were blocked for 1 h at 37°C in 0.01 M phosphate-buffered saline (PBS) containing 10% normal goat serum plus 0.3% Triton X-100. The sections were incubated in primary rabbit antibody for PICK1 (1:500) [14] for 48 h at 4°C. The specificity and selectivity of this antibody were reported previously [16]. The sections were finally incubated in biotinylated goat anti-rabbit IgG (1:200; Vector Laboratories, Burlingame, CA) for 1 h at 37°C followed by avidin-biotin-peroxidase complex (1:100; Vector) for 1 h at 37°C. The immune reaction product was visualized by catalysis of 3,3-diaminobenzidine by horseradish peroxidase in the presence of 0.01% H_2_O_2_.

Double-immunofluorescence histochemistry for PICK1/calcitonin gene-related peptide (CGRP) and for PICK1/NeuN was performed on the second and third sets of sections, respectively. Briefly, the sections were incubated sequentially with a mixture of rabbit polyclonal anti-PICK1 (1:500) and mouse monoclonal antibody to CGRP (1:200; Sigma, St. Louis, MO) or mouse monoclonal anti-NeuN (1:600; Chemicon, Temecula, CA) overnight at 4°C and a mixture of goat anti-rabbit IgG conjugated with Cy3 and donkey anti-mouse IgG conjugated with Cy2 (1:200; Jackson ImmunoResearch, West Grove, PA) for 1 h at room temperature. Double-fluorescence histochemistry for PICK1/biotinylated isolectin (IB)4 was performed on the fourth set of sections. Briefly, the sections were incubated sequentially with a mixture of IB4 (1:100; Sigma) and rabbit polyclonal anti-PICK1 (1:500) overnight at 4°C and a mixture of goat anti-rabbit IgG conjugated with Cy3 and Cy2-labeled avidin (1:200, Sigma) for 1 h at room temperature. In control double-labeling experiments, either of the primary antibodies was omitted or replaced with normal IgG or serum. All immunofluorescence-labeled sections were rinsed in 0.01 M PBS and mounted onto gelatin-coated glass slides. Cover slips were applied with a mixture of 50% glycerin and 2.5% triethylene diamine in 0.01 M PBS. The sections from double-labeling experiments were further observed with a confocal laser-scanning microscope by using laser excitation lines of 443-488 nm with appropriate emission filter for Cy3 (550-570 nm) or for Cy2 (492-510 nm).

For size distribution analysis of PICK1-positive cells within the L_5 _DRG, two sections were randomly selected from the first set of sections of each animal. All labeled and unlabeled cells with nuclei were counted. Cell profiles were outlined and cell area was calculated with the imaging software Image-Pro Plus (Media Cybernetics, Silver Spring, MD). For the analysis of PICK1/CGRP and PICK1/IB4 double-labeling within the L_5 _DRG, two sections each were randomly selected from the second, third, and fourth set of sections of each animal. Images were captured directly off the confocal laser-scanning microscope at 20 × objective magnification. Single-labeled (PICK1, CGRP, NeuN, or IB4) and double-labeled (PICK1/CGRP, PICK1/NeuN, or PICK1/IB4) neurons were counted, respectively.

### Post-embedding immunogold labeling and electron microscopy

Post-embedding immunogold labeling was carried out as described previously [6,12]. Briefly, WT mice (n = 2) were perfused transcardially with 4% paraformaldehyde + 0.5% glutaraldehyde. Cryoprotected sections from the L_5 _superficial dorsal horns were frozen in Leica CPC and freeze-substituted into Lowicryl HM-20 in a Leica AFS freeze substitution instrument. Ultrathin sections were labeled with polyclonal rabbit antibody for PICK1. Controls for specificity and selectivity of these antibodies were performed as described previously [16,20]. Areas for study were selected at random from the superficial dorsal horn at low magnification (i.e., synapses not visible) and then micrographs of synapses were taken at high magnification.

### Subcellular fractionation of proteins

Biochemical fractionation was carried out according to previous studies with minor modification [6,12,19]. The mice (10 WT and 10 KO mice/time point) were sacrificed by decapitation, and the tissues from L_4 _and L_5 _segments were dissected. The ipsilateral spinal cord was separated from the contralateral spinal cord under a surgical microscope and collected. Ipsilateral L_4 _or L_5 _spinal cords from two mice were pooled together and homogenized in homogenization buffer (50 mM Tris-HCl, 0.1 mM EDTA, 0.1 mM EGTA, 1 mM phenymethylsulfonyl fluoride, 1 μM leupeptin, 2 μM pepstain A). After the homogenate was centrifuged at 1,000 × *g *for 20 min at 4°C, the supernatant (S1, total soluble fraction) was collected and the pellet (P1, nuclei and debris fraction) discarded. The supernatant was centrifuged at 10,000 × *g *for 20 min to produce a pellet (P2) and supernatant (S2). The P2 was lysed hypo-osmotically in water and centrifuged at 25,000 × *g *to produce a final pellet (P3). The S2 was considered to be the crude cytosolic fraction and the P3 the crude synaptosomal membrane fraction.

### Western blotting analysis

After protein concentration was measured, proteins (30 μg) were heated for 5 min at 98°C and loaded onto 4% stacking/7.5% separating SDS-polyacrylamide gels for protein separation. The protein was then electrophoretically transferred onto a nitrocellulose membrane. The membrane was blocked with 3% nonfat dry milk and subsequently incubated for 1 h with polyclonal rabbit primary antibody for PICK1 (1:1,000), GluR2 (1:500; Upstate/CHEMICON, Temecula, CA), PKCα (1:1,000; Santa Cruz Biotechnology, Santa Cruz, CA), acid-sensing ion channel (ASIC) 1 (1:1000; Santa Cruz), ASIC2 (1:1000; Santa Cruz), or *N*-cadherin (1:1,000; BD Biosciences, Palo Alto, CA), and monoclonal mouse primary antibody for β-actin (1:3,000; Santa Cruz Biotechnology). The proteins were detected by horseradish peroxidase-conjugated anti-rabbit or anti-mouse secondary antibodies and visualized with chemiluminescence reagents provided with the ECL kit (Amersham Pharmacia Biotech, Piscataway, NJ) and exposure to film. The intensity of blots was quantified with densitometry. The blot density from naïve animals was set as 100%.

### Statistical Analysis

The results from the behavioral tests, Western blotting, and immunohistochemistry were analyzed statistically with a one-way or two-way analysis of variance (ANOVA). Data are presented as means ± SEM. When ANOVA showed significant difference, pairwise comparisons between means were tested by the *post hoc *Tukey method. Significance was set at *P *< 0.05. The statistical software package SigmaStat (Systat, San Jose, CA) was used to perform all statistical analyses.

## Results

**Figure 1:**
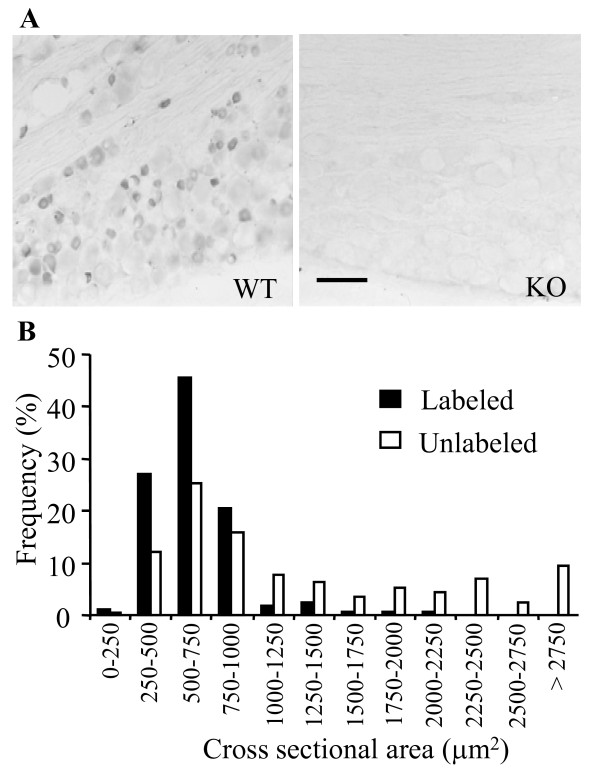
**Localization and distribution of PICK1 immunoreactivity in the dorsal root ganglion (DRG)**. **A**, Distribution of PICK1 immunoreactivity in the DRG cells of wild-type (WT; left) and PICK1 knockout (KO, right) mice. No immunoreactivity for PICK1 was observed in the DRG of PICK1 KO mice. Scale bar: 200 μm. **B**, Histogram showing the frequency of PICK1-positive and -negative somata in the DRG of WT mice by cross-sectional area.

**Figure 2:**
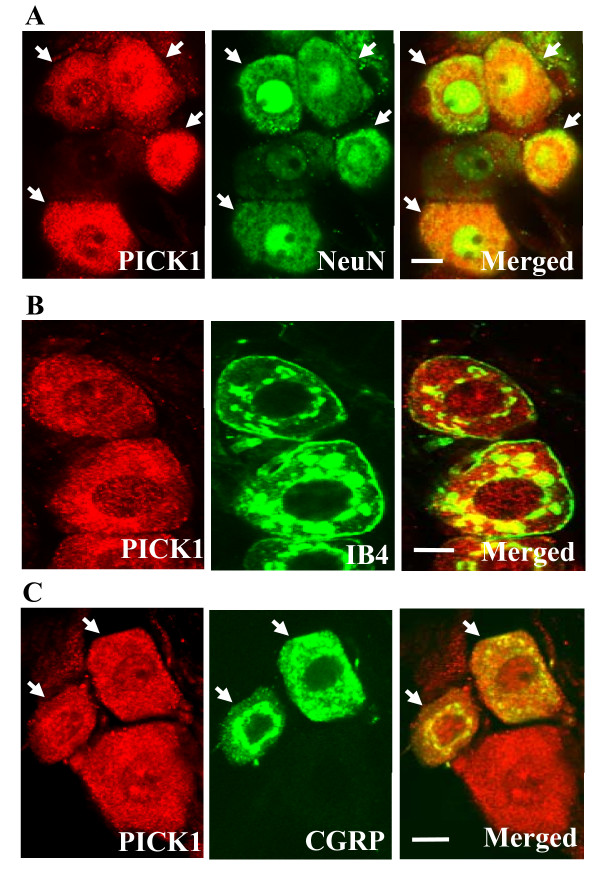
**PICK1 immunoreactivity in the DRG neurons and colocalization with IB4 binding and CGRP**. Digital images were taken with a confocal laser-scanning microscope. **A**, Colocalization of PICK1 and NeuN. **B**, Colocalization of PICK1 and IB4 binding. **C**, Colocalization of PICK1 and CGRP. Scale bars: 5 μm.

PICK1-positive cells were observed in the DRG of WT mice, but not of PICK1 KO mice (Figure 1A). Most were small, ranging from 250 to 750 μm^2 ^in surface area (Figure 1B). Some medium-size DRG cells were also PICK1-positive (Figure 1B). In contrast to small DRG cells, the population of large-diameter DRG cells mostly lacked PICK1. Approximately 29.7% of the 1124 DRG cells counted were PICK1-positive. Double-labeling studies showed that PICK1 was colocalized exclusively with NeuN, a marker for neuronal nuclei [21], but was absent from glia (Figure 2A). We further identified the cytochemical characteristics of PICK1-positive neurons in the DRG. Approximately 62.8% (211/336) of PICK1-positive neurons were positive for IB4, a marker for small DRG non-peptidergic neurons [21], and approximately 26.9% (125/465) of PICK1-positive neurons were positive for CGRP, a marker for small DRG peptidergic neurons [21].

**Figure 3:**
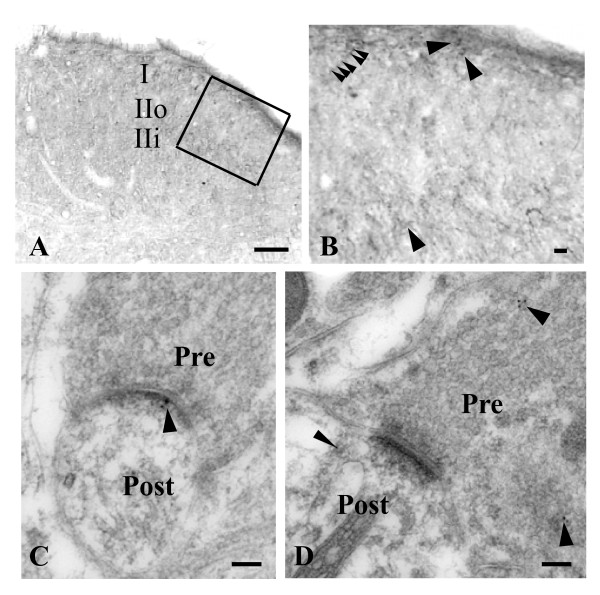
**Localization and distribution of PICK1 immunoreactivity in the spinal cord**. **A**, PICK1 immunoreactivity in the dorsal horn was strongest in lamina I and inner lamina II (IIi). IIo: outer lamina II. **B**, High magnification of the outlined region from A. PICK1-positive somata (arrows), punctation, and patches (arrow heads) were observed. **C**, Immunogold labeling for PICK1 is prominent in the postsynaptic density (arrow). **D**, Immunogold labeling for PICK1 is prominent in the presynaptic terminal (arrows) and postsynaptic dendrite (arrowhead). Pre: presynaptic. Post: postsynaptic. Scale bars: 100 μm in A, 5 μm in B, 100 nm in C and D.

PICK1 protein was also observed in the spinal cord. PICK1 immunoreactivity was distributed at a higher density in the superficial dorsal horn than in other regions of spinal cord, particularly in the lamina I and inner lamina II (Figure 3A). Under high magnification, immunoreactivity to PICK1 was observed mostly as punctate or patch immunostaining (Figure 3B). A few PICK1-positive cell bodies were seen in dorsal horn (Figure 3B). Under electron microcopy, immunogold-labeling for PICK1 was prominent in the postsynaptic density, dendrites, and presynaptic terminals in the superficial dorsal horn (Figure 3C and 3D).

**Figure 4:**
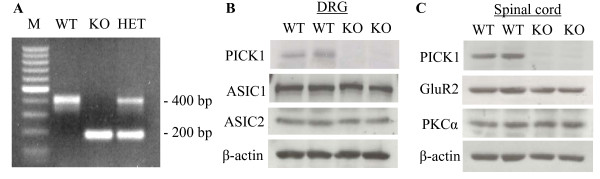
**Biochemical characterization of PICK1 knockout (KO) mice**. **A**, PCR analysis of tail DNA from wild-type (WT), heterozygous (HET), and PICK1 KO mice. M: molecular weight standard. **B**, Western blot analysis shows the expression of PICK1 protein and its interacting proteins ASIC1 and ASIC2 in the DRG from WT and PICK1 KO mice. **C**, Western blot analysis shows the expression of PICK1 and its interacting proteins GluR2 and PKCα in the spinal cord from WT and PICK1 KO mice.

We further used behavioral testing to determine whether PICK1 participated in transmission and modulation of nociceptive information. Because PICK1 is a scaffolding protein without any receptor-like or enzyme-like activities, we used a genomic strategy in which the PICK1 gene was mutated. As reported previously [16], PICK1 KO male and female mice are viable with normal appearance. The genomic status of each mouse was checked with PCR (Figure 4A), and the expression of PICK1 protein in the DRG and spinal cord also was verified by Western blotting analysis and immunohistochemistry. As expected, PICK1 was not detected in the DRG or spinal cord of the PICK1 KO mice (Figures 1A and 4B). In addition, the expression of PICK1 interacting proteins was evaluated in the DRG and spinal cord. Western blotting analysis revealed that the levels of interacting proteins ASIC1 and ASIC2 in the DRG (Figure 4B) and the levels of interacting proteins GluR2 and PKCα in the spinal cord (Figure 4C) were normal in the PICK1 KO mice. The DRG and spinal cord cell architecture appeared normal in PICK1 KO mice (data not shown). These results suggest that the targeted disruption of PICK1 gene does not produce any detectable abnormality of DRG or spinal cord anatomical structure or protein expression except for the loss of PICK1 protein.

**Figure 5:**
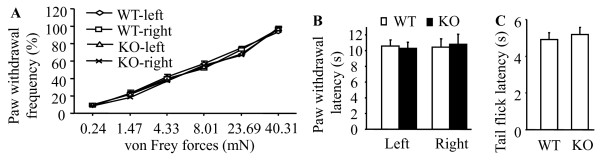
**PICK1 knockout (KO) preserves acute pain**. **A**, Baseline paw withdrawal frequencies in response to various forces of von Frey monofilaments in naïve wild-type (WT) and PICK1 KO mice. **B**, Baseline paw withdrawal latencies in response to heat stimulation in naïve WT and PICK1 KO mice. **C**, Baseline tail-flick latencies in response to heat stimulation in naïve WT and PICK1 KO mice.

We first examined whether the deletion of PICK1 affected acute nociception. Acute mechanical sensitivity was assessed by response frequencies of paw withdrawal to mechanical stimulation elicited by different forces of von Frey filaments. Acute thermal sensitivity was evaluated by high-intensity radiant heat applied to the plantar sides of left and right hind paws and the tail. We found that paw withdrawal responses of PICK1 KO mice to acute mechanical (Figure 5A) and thermal stimuli (Figure 5B) as well as tail flick response to acute thermal stimulation (Figure 5C) were similar to those of WT mice. These data indicate that transmission of acute pain messages is preserved in PICK1 KO mice.

**Figure 6:**
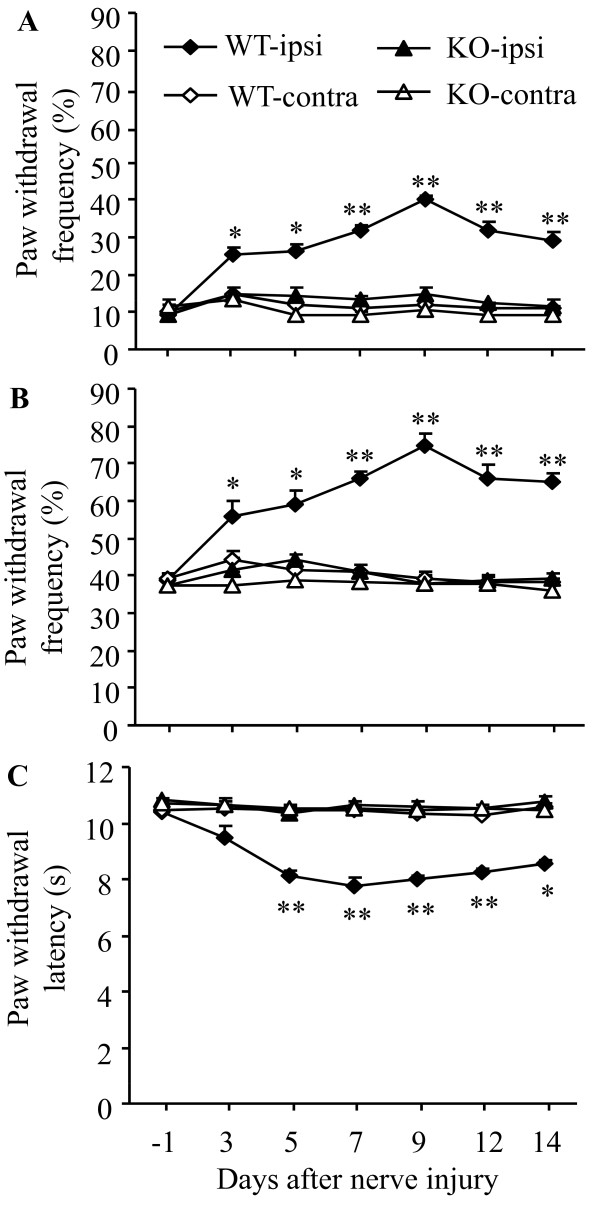
**PICK1 knockout (KO) mice exhibit impaired neuropathic pain**. L_5 _SNL significantly increased paw withdrawal frequencies in response to 0.24 mN (low intensity; **A**) and 4.33 mN (moderate intensity; **B**) mechanical stimuli and significantly decreased paw withdrawal latency in response to heat stimulation (**C**) on the ipsilateral (ipsi), but not contralateral (contra), side in wild-type (WT) mice. PICK1 KO mice failed to exhibit any changes in paw withdrawal frequencies or latencies during the observation period. * *P *< 0.05, ** *P *< 0.01 *vs *the corresponding time points in PICK1 KO mice on the ipsilateral side.

Next, we determined the role of PICK1 in neuropathic pain. Consistent with our previous studies [6,22], L_5 _SNL produced long-term mechanical and thermal pain hypersensitivity on the hind paw ipsilateral to nerve injury in WT mice. The application of 0.24 mN (low intensity) and 4.33 mN (moderate intensity) von Frey filaments to the plantar side of the ipsilateral hind paw elicited paw withdrawal frequencies that were significantly greater than those at pre-injury baseline values. This mechanical pain hypersensitivity was detectable on day 3, reached a peak level between days 7 and 9, and persisted for at least 14 days post-nerve injury (Figure 6A and 6B). Likewise, paw withdrawal latencies in response to application of heat to the plantar side of the ipsilateral hind paw were markedly decreased from the pre-injury baseline values. This thermal pain hypersensitivity developed within 5 days and persisted for at least 14 days (Figure 6C). The baseline withdrawal responses to mechanical and thermal stimuli were similar in WT and PICK1 KO mice as described above (Figure 6), but PICK1 KO mice did not display mechanical or thermal pain hypersensitivity following spinal nerve injury. Paw withdrawal responses to mechanical and thermal stimuli were unchanged compared to the baseline levels in the PICK1 KO mice (*P *> 0.05; Figure 6). As expected, paw withdrawal responses to mechanical and thermal stimuli were not altered in the contralateral hind paw in either WT or PICK1 KO mice after spinal nerve injury (Figure 6).

Because locomotor function is involved in paw withdrawal reflex response, we tested whether the deletion of PICK1 affected locomotor functions. We recorded the scores for three gross reflexes (placing, grasping, and righting) in WT and PICK1 KO mice. We found that PICK1 KO mice performed the same as their WT littermates for the three reflexes (Table 1) and did not exhibit gross motor coordination defects. The results suggest that gross locomotor function is not impaired by PICK1 deficiency and cannot account for the observed deficiency of nerve injury-induced pain behaviors.

**Table 1:** Mean (SEM) changes in locomotor test

	Placing	Grasping	Righting
WT	5 (0)	5 (0)	5 (0)
PICK1 KO	5 (0)	5 (0)	5 (0)

N = 5, 5 trials

**Figure 7:**
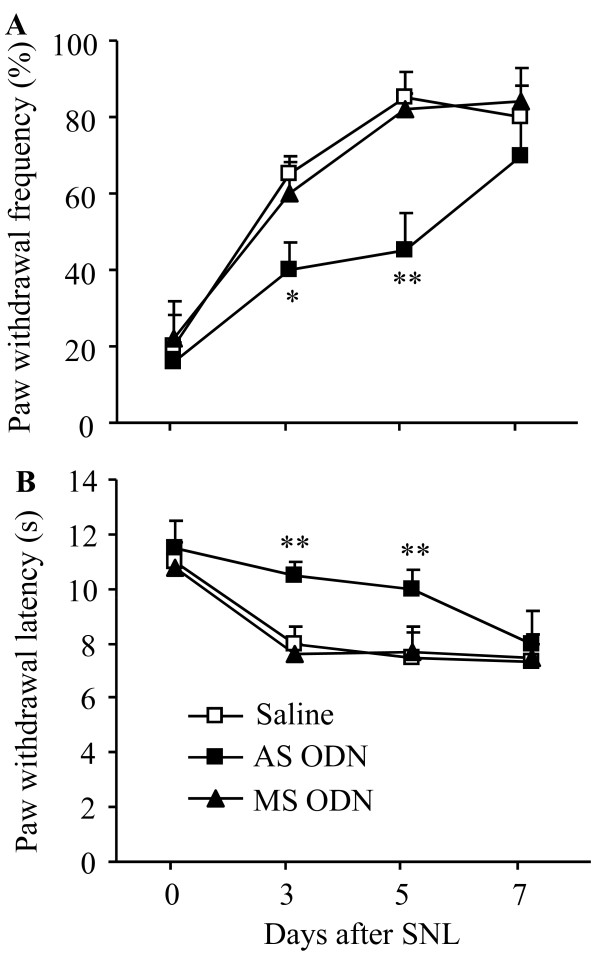
**PICK1 knockdown blunts neuropathic pain**. L_5 _spinal nerve ligation (SNL) led to a significant increase in paw withdrawal frequency in response to 8.01 mN mechanical stimulation (**A**) and a marked decrease in paw withdrawal latency in response to heat stimulation (**B**) on the ipsilateral side in the saline-treated rats. Intrathecal administration of PICK1 antisense (AS) oligodeoxynucleotide (ODN, 10 μg) delayed the development of both mechanical and thermal pain hypersensitivities on days 3 and 5 post-SNL. No significant differences in paw withdrawal frequencies or latencies were observed between rats treated with saline and those treated with missense (MS) ODN (10 μg). ** *P *< 0.01 vs the same time points in the saline-treated group.

PICK1 genetic KO mice, like other congenital KO mice, might undergo unknown compensatory changes in the nervous system during development. Such changes might affect animal behaviors. To further confirm the role of PICK1 in neuropathic pain, we used PICK1 AS ODN to acutely and transiently knock down PICK1 in spinal cord and DRG of rats. Our previous study showed that i.th. administration of PICK1 AS ODN (10 μg), but not MS ODN (10 μg), selectively and significantly reduced PICK1 expression in spinal cord and DRG [14]. This reduction did not affect basal mechanical and thermal responses or locomotor function, but it did attenuate CFA-induced mechanical and thermal pain hypersensitivities [14]. Here, we found that after i.th. injection of 10 μg PICK1 AS ODN, mechanical and thermal pain hypersensitivities were markedly blunted until day 7 post-SNL (Figure 7). Significant differences in paw withdrawal frequencies and latencies were observed between the AS group (n = 5) and saline group (n = 5; *P *< 0.01). As expected, 10 μg of MS ODN did not affect the development of nerve injury-induced mechanical and thermal pain hypersensitivities (*P *> 0.05, n = 5; Figure 7).

**Figure 8:**
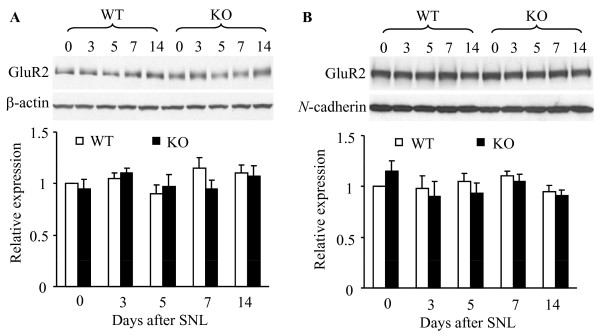
**L_5 _spinal nerve ligation (SNL) does not induce GluR2 internalization in dorsal horns from wild-type (WT) mice**. **A**, GluR2 levels in the cytosolic fraction from the ipsilateral dorsal horns of WT and PICK1 knockout (KO) mice at different time points after L_5 _SNL. Top: Representative Western blots. Bottom: Statistical summary of the densitometric analysis expressed relative to naïve (0) mice after normalization to corresponding β-actin. **B**, GluR2 levels in the synaptosomal membrane fraction from the ipsilateral dorsal horn of WT and PICK1 KO mice at different time points after L_5 _SNL. Top: Representative Western blots. Bottom: Statistical summary of the densitometric analysis expressed relative to naïve (0) mice after normalization to corresponding *N*-cadherin.

Results from our previous study suggested that PICK1-mediated dorsal horn GluR2 internalization contributes to the maintenance of CFA-induced chronic inflammatory pain. Therefore, we examined whether a similar process might occur in neuropathic pain. Crude cytosolic and synaptosomal membrane protein fractions were separated by using differential centrifugation. Consistent with our previous studies [11,12,19], a plasma membrane-specific protein, *N*-cadherin, was detected highly in the membrane fractions, but not in the cytosolic fraction (data not shown), indicating that the fractionation procedure effectively separated cytosolic proteins from synaptosomal membrane proteins. We found that L_5 _SNL did not change the level of GluR2 in either the cytosolic or the synaptosomal fraction from the ipsilateral L_4 _(data not shown) and L_5 _(Figure 8) dorsal horns of WT and PICK1 KO mice during the observation period. These findings indicate that peripheral nerve injury does not induce GluR2 internalization in dorsal horn.

## Discussion

Neuropathic pain is a chronic condition that affects millions of people worldwide. Current treatments are often inadequate, ineffective, or produce potentially severe adverse effects. Therefore, the search for novel therapeutic and preventive strategies is essential. Here, we report that PICK1, a PDZ domain-containing scaffolding protein, might be required for the induction of nerve injury-induced neuropathic pain, suggesting that PICK1 may be a new target for the prevention and/or treatment of this disorder.

The present study demonstrated that PICK1 was expressed in two major pain-related regions, DRG and dorsal horn. We found that approximately 30% of DRG neurons were positive for PICK1 and that most of such neurons were small. Both peptidergic and non-peptidergic neurons were represented. In the dorsal horn, PICK1-positive terminals, fibers, and cell bodies were concentrated on the superficial dorsal horn, especially in lamina I and inner lamina II. Under electron microscopy, immunogold labeling for PICK1 was associated with the postsynaptic density in the dendrites of the superficial dorsal horn neurons. In line with this finding, we previously showed that PICK1 was expressed more highly in the dorsal horn than in the ventral horn or DRG [14]. Abundant expression of PICK1 in small DRG neurons and superficial dorsal horn neurons suggests that it might participate in transmission and modulation of nociceptive information.

PICK1 plays distinct roles in acute pain and neuropathic pain. The present study showed intact response to noxious mechanical and thermal stimuli in naïve PICK1 KO mice, indicating that PICK1 might not be involved in acute pain transmission. In contrast, PICK1 knockout abolished nerve injury-induced pain hypersensitivity during the development and maintenance periods. Moreover, PICK1 knockdown in spinal cord and DRG significantly attenuated nerve injury-induced development of pain hypersensitivity. These findings suggest that PICK1 in spinal cord and DRG contributes to neuropathic pain. Our previous study demonstrated that spinal PICK1 deficiency also attenuated pain hypersensitivity during the maintenance period of CFA-induced inflammatory pain [14]. Moreover, we found that this effect might be attributable to reduction of NMDA receptor/PKC-triggered dorsal horn GluR2 internalization in CFA-induced inflammatory pain maintenance [12-14]. We expected to observe this mechanism in neuropathic pain as well. However, we detected no significant change in the level of GluR2 in cytosolic or synaptosomal membrane fractions from dorsal horn of WT or PICK1 KO mice after L_5 _SNL. These results indicate that SNL-induced nerve injury input, unlike CFA-induced inflammatory input, does not promote dorsal horn GluR2 internalization. Hence, PICK1 may participate in neuropathic pain development via other potential mechanisms, even as it binds to its partners PKCα and GluR2.

In addition to interacting with GluR2, PICK1 interacts specifically with the C-termini of ASIC1 and ASIC2 in central neurons via its PDZ domain [23,24]. ASIC1 is expressed predominantly in small-diameter DRG neurons, whereas ASIC2 is localized to the plasma membrane of small-, medium-, and large-diameter cells of the DRG [25-27]. Disrupting the mouse ASIC2 gene markedly reduces the sensitivity of a specific component of mechanosensation [28]. Moreover, PICK1 potentiates PKA phosphorylation of ASIC1 and increases ASIC2 current by enhancing its phosphorylation by PKC [29]. It seems that the anti-hyperalgesic effect caused by PICK1 deficiency in neuropathic pain might be due to a reduction in function of DRG ASIC1 and ASIC2.

PICK1's PDZ domain also binds to metabotropic glutamate receptor subunit 7 (mGluR7) in central neurons. An inhibitory presynaptic receptor, mGluR7 is expressed in the DRG neurons and primary afferent terminals [30,31]. It is activated under neuropathic pain conditions, but not under normal conditions [32-34]. Because PICK1 inhibits PKC phosphorylation of mGluR7 [9,35,36], it is possible that PICK1 deficiency causes a loss of inhibition of mGluR7 phosphorylation and potentiates mGluR7 activation. Activated mGluR7 would then inhibit glutamate release from primary afferents and result in an antinociceptive effect in neuropathic pain. The possibility that DRG PICK1 might contribute to neuropathic pain by regulating ASIC1, ASIC2, and mGluR7 functions remains to be further confirmed. It is noteworthy that during neuropathic pain PICK1 still interacts with other proteins, such as Eph receptor tyrosine kinase [37], dopamine transporter [38], and class I ADP-ribosylation factors [39]. Whether these interactions are involved in neuropathic pain is unknown.

In summary, we showed that PICK1 was expressed in the DRG and superficial dorsal horn neurons. Behavioral studies demonstrated that knockout of PICK1 abolished pain hypersensitivity during nerve injury-induced neuropathic pain development and maintenance, without affecting acute pain or locomotor function. Although the mechanism of this antinociceptive effect in neuropathic pain is still unclear, PICK1 appears to be a potential target for prevention and/or treatment of this disorder.

## Competing interests

The authors declare that they have no competing interests.

## Authors' contributions

WW participated in design of the study, carried out surgery, SNL model, behavioral testing, RT-PCR, immunohistochemistry, and Western blot experiments, and wrote a draft of the manuscript. RSP was involved in immunogold labeling and electron microscopy observation. KT, JX, and RLH were involved in generating KO mice. YQL and RLH participated in critical review the manuscript. YXT and MY contributed to the concept and design of the study, the data analysis and interpretation, and critical review the manuscript. All authors have read and approved the final manuscript.
